# Effect of Ultrasonic Pretreatment on Flocculation Filtration of Low-Rank Coal Slurry

**DOI:** 10.3390/molecules27196460

**Published:** 2022-09-30

**Authors:** Aosheng Yang, Yinfei Liao, Maoyan An, Yijun Cao, Zhe Yang, Hourui Ren, Hailong Su, Qiqi Zou, Luojian Chen

**Affiliations:** 1 National Engineering Research Center of Coal Preparation and Purification, China University of Mining and Technology, Xuzhou 221116, China; 2 School of Chemical Engineering and Technology, China University of Mining and Technology, Xuzhou 221116, China; 3 School of Transportation Engineering, Jiangsu Vocational Institute of Architectural Technology, Xuzhou 221116, China; 4 Jiangsu Engn Lab Biomass Resources Comprehens Uti, Jiangsu Vocational Institute of Architectural Technology, Xuzhou 221116, China

**Keywords:** ultrasonic pretreatment, low-rank coal, flocculation filtration, L-F NMR, filter cake porosity

## Abstract

The efficient filtration of low-rank coal (LRC) slurry was significantly beneficial to the production process of wet coal beneficiation. However, relatively few studies have been reported on novel pretreatment methods for the efficient filtration of LRC slurry. In this paper, the mechanism of ultrasonic pretreatment to promote flocculation and filtration of slurry was studied. The hydrophobic variation of the slurry surface was measured by contact angle and XPS. The flocculation properties of slurry were characterized using zeta potential and FBRM. The effects of filter cake porosity and ultrasonic pretreatment on slurry filtration resistance were calculated by L-F NMR and Darcy’s theory. The results showed that the ultrasonic pretreatment promoted the flocculation and filtration performance of LRC slurry, increased the filtration rate, and decreased the cake moisture content. Meanwhile, the contact angle of LRC increased significantly from 50.1° to 67.8° after ultrasonic pretreatment, and the surface tension of the filtrate decreased from 69.5 to 53.31 mN/m. Ultrasonic pretreatment reduced the absolute value of the zeta potential of coal slurry from 24.8 to 21.0 mV, and the average chord length of flocs increased from 5–10 μm to 25–30 μm, thus weakening the electrostatic repulsion between coals to promote floc formation. In addition, the pore tests and filtration theory calculations showed that the ultrasonic pretreatment significantly improved the permeability of the filter cake to water and reduced the resistance to slurry during filtration. In particular, the mesopore porosity increased by 9.18%, and the permeability increased by 2.937 × 10^8^ m^2^. Therefore, this contributed to the reduction of slurry filtration resistance. This research provides an efficient method for promoting the efficient filtration of slurry.

## 1. Introduction

Low-rank coal (LRC) has abundant reserves worldwide and is relatively inexpensive to extract, which makes LRC an important part of energy production [1,2]. With the deterioration of the geological conditions of coal seams and the continuous development of mechanized large-scale mining techniques, the fine LRC accounts for approximately 20 wt.% of the raw coal [3,4]. At present, wet coal beneficiation technology is widely used. The filtration and dewatering process is an important part of wet coal beneficiation. The effective filtration of LRC slurry not only improved the product quality, but also facilitated the water recycling and protected the environment of the mine area [5].

However, it has been found that fine coal is difficult to filter and dewater during the production of coal processing plants. It was found that fine particle coals tend to form filter cakes with a fine pore structure during filtration, whereas fine pores have strong capillary forces that adversely affect the filtration process [6,7]. Meanwhile, the clay minerals contained in LRC slurry tend to form thick hydration films in water, which not only increases the filtration resistance but also decreases the filtration rate. In addition, particle size, surface wettability, and ash content have an effect on the filtration of slurry [5,8].

To enhance the filtration performance of LRC slurry, scholars conducted extensive research. The filter aids were widely concerned because the source was widespread, the production process was simple, and the filtration effect was good. Quaternary ammonium surfactants could enhance particle surface hydrophobicity and reduce coal surface charge, thus facilitating the slurry filtration [9]. Stearyl trimethyl ammonium chloride is a cationic surfactant, and its nitrogen atoms adsorb on the particles, orienting their alkyl chains towards water, thus weakening the particle–water interaction [10]. The ionic flocculant in slurry has strong affinity for the surface of coal particles and high adsorption density, and it could reduce the surface potential of the particles [11]. Meanwhile, flocculants can promote floc formation and remove free water from the larger pores of the filter cake [12,13]. In addition, a new microbial flocculant has been discovered, and it could improve floc size and promote sludge filtration [14]. In conclusion, current research has focused on the effect of reagents on particle performance or the development of new filter aids. However, there are relatively few studies on the use of novel treatment methods in LRC slurry filtration.

In recent years, ultrasonic treatment has been used in mineral processing, sludge filtration, and the chemical industry [15,16,17]. The literate indicates that the dispersion of reagents in the suspension is enhanced using ultrasonic treatment, reduces the electrostatic repulsion between particles, and promotes the increase of floc size [18]. Meanwhile, the adsorption tests proved that ultrasonic treatment could enhance the adsorption of reagents [19,20]. In addition, ultrasonic treatment was found to improve the looseness of the cake and promote the formation of cake macropores in municipal sludge, thus facilitating sludge filtration [21]. Although ultrasonic treatment has been widely applied to mineral processing, sludge treatment, and the chemical industry, it has rarely been applied to LRC slurry filtration.

In this paper, the effect of ultrasonic pretreatment on the flocculation filtration performance of LRC slurry was investigated. Contact angle, XPS, and FBRM were used to measure the property changes of coal slurry after ultrasonic pretreatment. Meanwhile, the changes of ultrasonic pretreatment on the electrostatic repulsion between LRC slurry particles and the rheological properties of the filtrate were analyzed. In addition, the effect of ultrasonic pretreatment on the porosity of the filter cake was determined. The slurry filtration resistance variation and filter cake permeability change were calculated using Darcy’s filtration theory. The results can provide a new pretreatment method to explore the efficient flocculation and filtration of LRC slurry.

## 2. Experiment and Methods

### 2.1. Materials

The coal samples of fine grain were taken from Yulin, Shaanxi Province, China. Prior to the test, the coal samples were washed with tap water. The raw coal was placed in a stirred tank, and after stirring and standing, the supernatant was removed. This process was repeated three times. The final product was then filtered, dried, and stored in sealed plastic sample bags as test samples. The industrial analysis was conducted by an industrial analyzer (5E-MAG6700, Kaiyuan Technology Development Co., Ltd., Changsha, China), as listed in Table 1.

**Figure 1:**
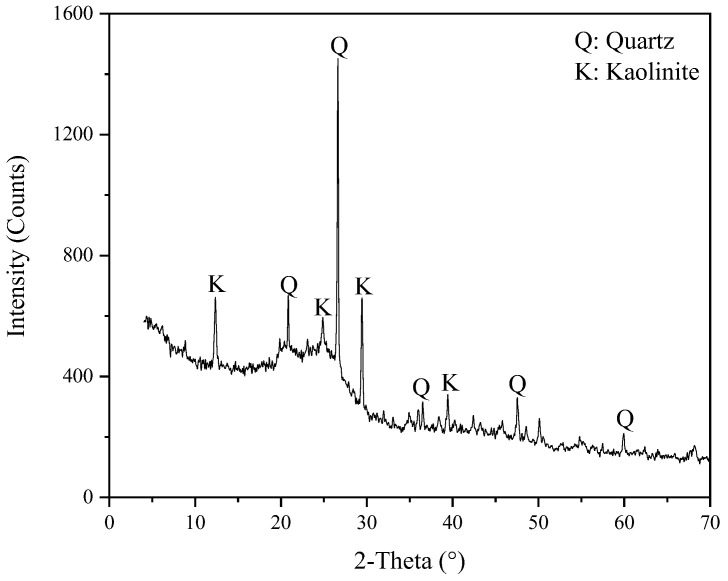
LRC sample of X-ray diffraction (XRD) analysis.

**Figure 2:**
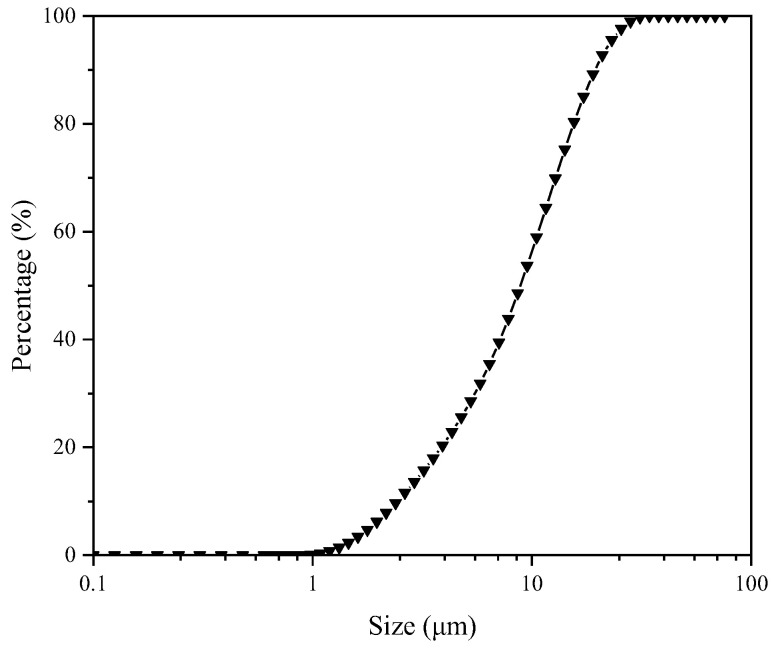
Particle size analysis of LRC sample.

The results showed that the ash content (Aad) and the moisture content (Mad) of the sample were 16.74% and 7.35%. The air-drying volatile matter (Vad) and the fixed carbon (FC) content were 38.34% and 54.67%. In order to investigate the types of minerals in the LRC slurry, X-ray diffraction analysis was performed. The result is shown in Figure 1. Quartz and kaolinite were the mineral impurities in the sample. A laser diffractometer (Malvern Mastersizer 2000, Malvern Panalytical, London, UK) was used to measure the particle size distribution of the sample particles, and the results of the particle size test was shown in Figure 2. The d_50_, d_80_, and d_90_ values of the coal samples were 8.617, 15.576, and 18.975 μm, which indicated that the coal samples were microfine samples.

Thoroughly mixed 40 g of dry coal samples with 160 mL of deionized water under a stirring speed of 500 r/min was used to prepare the slurry for the experiments. The salinity value of the coal slurry sample was about 1000 mg/L. The volume of the prepared coal slurry sample was about 200 mL. The coal slurry and deionized water were used in all performance analyses.

Analytical-grade anionic polyacrylamide (APAM) was used with a weight of 12 million daltons, a hydrolysis degree of 10–35%, and a molecular formula of (C_3_H_5_NO)_n_. APAM was taken from Aladdin Chemicals Ltd., China.

### 2.2. Methods

#### 2.2.1. Ultrasonic Pretreatment

**Figure 3:**
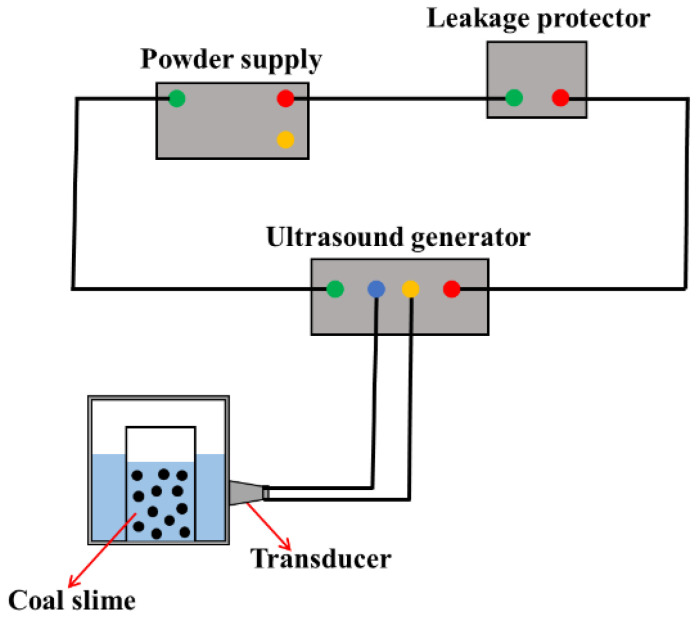
Schematic of the ultrasound equipment.

The schematic diagram of the ultrasonic pretreatment system is shown in Figure 3. The ultrasound action system consisted of an ultrasound generator (SL-650SD, Nanjing Hanzhou Technology Co., Ltd., Nanjing, China) and several ultrasound transducers. The frequencies of the selected transducers were 10, 20, 30, 40, and 50 KHz. The maximum output power of the ultrasonic generator was 1100 W, and the maximum output power of several ultrasonic probes was 100 W. The ultrasound amplitude ranged from about 80 to 100 μm. The bath was rectangular in cross section, with a length of 200 mm, a width of 150 mm, and a height of 130 mm. During the ultrasonic pretreatment, the temperature change in the bath was about 2 °C. In the ultrasonic pretreatment, first, APAM was added into the slurry suspension and stirred for 5 min, and then the slurry was placed in the ultrasonic system for the corresponding time.

#### 2.2.2. Flocculation and Filtration Tests

**Figure 4:**
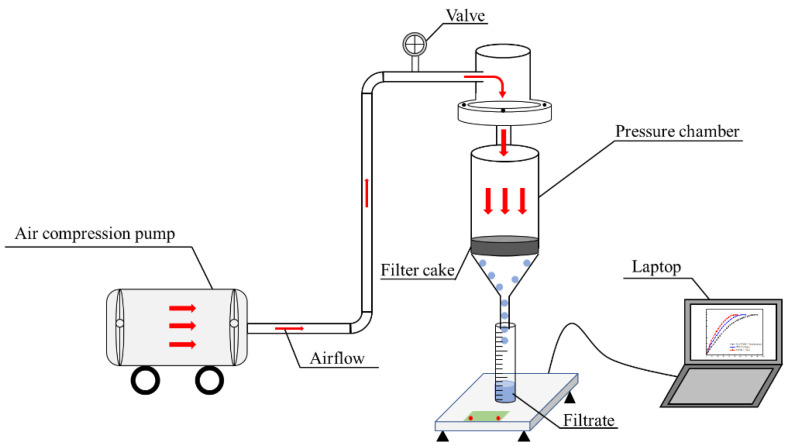
Schematic positive pressure filtration equipment.

The LRC slurry filtration equipment is illustrated in Figure 4. The pressure of the filtration equipment was set at 0.2 MPa, and the slurry was quickly poured into the filtration equipment at the beginning of the filtration experiment. The cake and filtrate were then collected, and the filtration time was recorded. The filtrate was collected as the filtration experiment proceeded. After filtration, the aqueous filter cake was collected and weighed. Afterwards, the filter cake was dried at 60 °C, and the weight of the dried filter cake was recorded. The method of filter cake moisture content calculation is given by Equation (1):

(1) $$M_{w}=\frac{M_{c}-M_{d}}{M_{c}}\times 100\%$$

where *M_w_* is the moisture of the filter cake, *M_c_* is the weight of the aqueous filter cake, and *M_d_* is the weight of the dried filter cake.

#### 2.2.3. Measurement of Coal Slurry Properties

The variation of the reagent on the functional group content of the LRC surface was measured and analyzed using XPS (ESCALAB 250Xi, Thermo Scientific, New York, NY, USA). The monochromatic X-ray source used for XPS was Al Kα radiation (hv = 1486.6 eV). For the XPS analysis, the take-off angle of the photoelectrons was 90°, and the spot size was 900 μm. The measured scanned spectra were recorded with a pass energy of 100 eV and an energy step of 1.00 eV. The high-resolution spectra were recorded with a pass energy of 20 eV and an energy step of 0.05 eV. This data processing (peak fitting) was performed using the Gasa XPS software. The binding energy was corrected by setting the C1s hydrocarbon (-CH2-CH2-bond) peak to 284.6 eV.

The contact angle size of the LRC slurry was measured by a DSA 100 (Kruse, Berlin, Germany). The coal sample was pressed by a press with a pressure of 50 t into a circular slurry body of about 2 mm thickness. Afterwards, the pressed coal piece was placed under a contact angle measuring instrument for measurement. The contact angle of the coal sample was obtained by computer analysis of the shape of the droplets on the coal piece.

The viscosity (*η*) of each sample was measured at a shear rate of 900 s^−1^. Approximately 5 mL of filtrate was collected using a viscometer (Haake, Berlin, Germany). Each sample was tested five times, and the average value was the viscosity of the filtrate.

The pH of the slurry was adjusted using NaOH and HCl solutions. The slurry was collected in a 50 mL centrifuge tube and then centrifuged at 800 r/min for 5 min to transfer the supernatant to a platinum electrode and measure the zeta potential at an ambient temperature of 20 °C. The test was repeated five times, and the average value was taken as the result.

**Figure 5:**
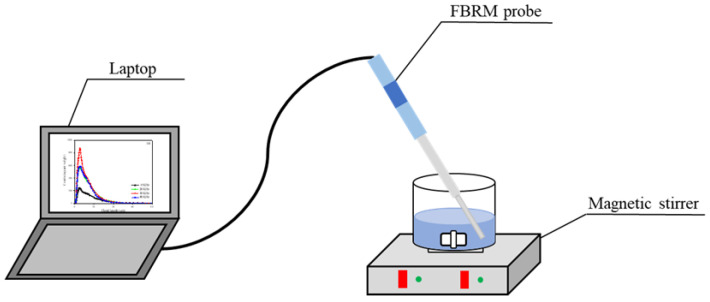
Diagram of the FBRM test system.

The focused beam reflectance measurement (FBRM, G400, Mettler Toledo, Columbus, OH, USA) system is shown in Figure 5. FBRM was a probe-based measurement method that inserted the instrument directly into the solution to study the change of particle size and counts over time, and allowed the measurement of the highly turbid sample [22,23]. In this study, FBRM was adopted to monitor the evolution of the floc size, amount, and micromorphology of the flocs online. The chord length of the square-weighted average diameter was used to characterize the size of the floc. A 200 mL beaker was filled with 0.5 g of mucilage and stirred at 500 r/min for 5 min. During this time, the counts and chord lengths of flocs were collected with the FBRM probe. The ICFBRM4.3 software was used to analyze the counts, chord lengths, and micromorphology of the flocs.

The surface tension was measured by K100 (Kruss, Berlin, Germany). The filtrate after slurry filtration was collected. The platinum plate was treated by burning with an alcohol lamp and then cooling to 20 °C. The value of the surface tension was the average of five measurements.

#### 2.2.4. Pore Measurements

The pore size distribution of the filter cake was measured by the L-F NMR instrument (NMRC12-010V, Neway Analytical Instrument Co., Ltd, Niumag, China). L-F NMR was a nondestructive and rapid technique for measuring the pore size distribution of solid. Nuclear magnetic resonance occurred when the hydrogen protons were activated by a static magnetic field and exposed to a second oscillating magnetic field. The pore size and pore throat distribution of the filter cake were indicated by assessing the intensity of hydrogen protons in water [24,25]. An amount of 1 g of sample taken from the center of the filter cake was put into the L-F NMR tube and sealed to prevent water loss during the pore testing. The resonance frequency of the equipment was 12.5 MHz. The temperature of the magnets of L-F NMR was kept constant at 32 °C [26].

#### 2.2.5. Darcy’s Filtration Theory Calculation

The total resistance during filter cake formation was the sum of the medium resistance (*R_m_*) and the filter cake resistance (*α*), which can be determined by the Darcy law and the Carman–Kozeny equation, as shown in Equation (2) [27]. The filter cake resistance and medium resistance were generated by the filter cake and the filter media in the filtration process, respectively. The filter cake resistance was mainly influenced by the properties of the filter cake. However, the medium resistance was mainly related to the properties of the filter media whose pores may be blocked by microfine slurry particles. Media resistance can be estimated in the early stages of coal slurry filtration when the filter media were not easily deformed by squeezing, after which they remained essentially constant with increasing filtration time [25,27]:

(2) $$\frac{t}{V}=\frac{\alpha \mu c}{2A^{2}\Delta P}V+\frac{R_{m}\mu }{A^{2}\Delta P}$$

where *t* is the filtration time in s, and *V* is the volume of the filtrate at time *t* in m^3^. The parameters *c*, *μ*, *A*, and Δ*P* represent the slurry concentration, absolute viscosity of water, cake area, and differential pressure, respectively. The values of the parameters required for Equation (2) are given in Table 2. The filter medium resistance (*R_m_*) and the filter cake resistance (*α*) in equation (2) showed that *t/V* was the dependent variable and *V* was the independent variable. Thus, a graph of *t/V* versus *V* permitted the calculation of the gradient and intercept, which can be used to calculate α and Rm. The permeability of the filter cake (*K*) can be calculated using Equation (3):

(3) $$K=\frac{1}{\alpha \left(1-\varepsilon \right)\rho_{s}}$$

where *ε* is the cake porosity, *ρ_s_* is the density of particles, and *K_0_* is the Kozeny constant, which is approximately equal to 5 for fixed or slowly moving beds [25].

## 3. Results and Discussion

### 3.1. The LRC Slurry Flocculation Filtration Results

**Figure 6:**
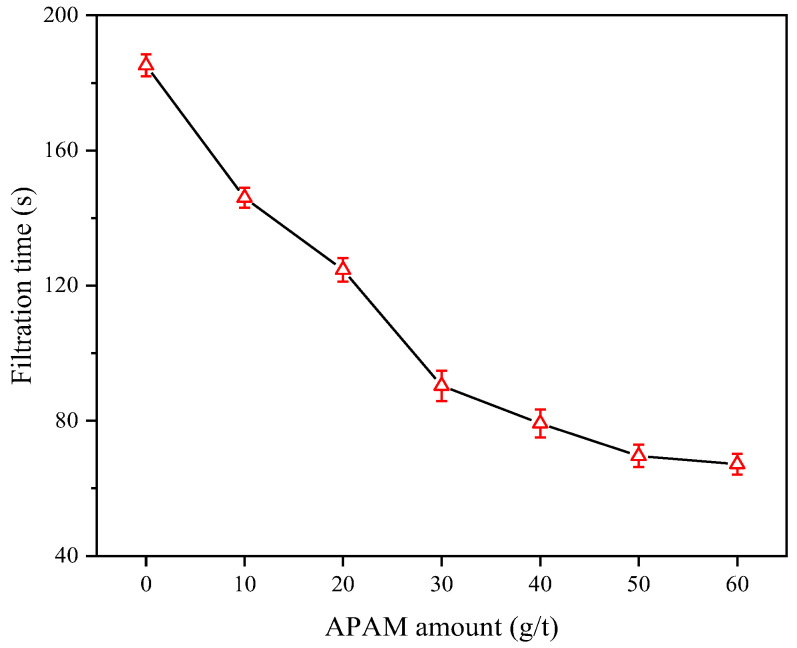
Filtration time with APAM dosage.

The flocculant dosage is critical to achieve efficient flocculation and filtration of slurry. Figure 6 illustrates the variation of the slurry filtration time with the increase in APAM dosage. The results indicated that the filtration time of coal slurry was gradually reduced with increased APAM dosage. When the APAM dosage was increased from 0 to 50 g/t, the slurry filtration time was decreased from 185.2 to 69.6 s. When the APAM dosage exceeded 50 g/t, the change of slurry filtration time tended to be stable.

**Figure 7:**
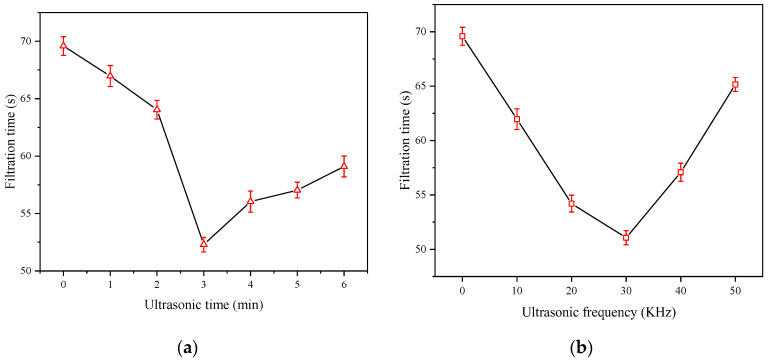
Effect of ultrasonic time (**a**) and ultrasonic frequency (**b**) on LRC slurry filtration time.

Figure 7a,b illustrates the effect of ultrasonic time and ultrasonic frequency on slurry filtration time. The slurry filtration time was 69.6 s without ultrasound pretreatment. When the slurry was pretreated by ultrasound for 3 min, the filtration time was decreased sharply to 52.3 s. On the other hand, when the ultrasonic frequency was increased to 30 KHz, the filtration time was decreased significantly to 51.1 s. The optimum amount of APAM was 50 g/t, and the best ultrasonic condition was an ultrasonic frequency of 30 KHz and an ultrasonic time of 3 min.

**Figure 8:**
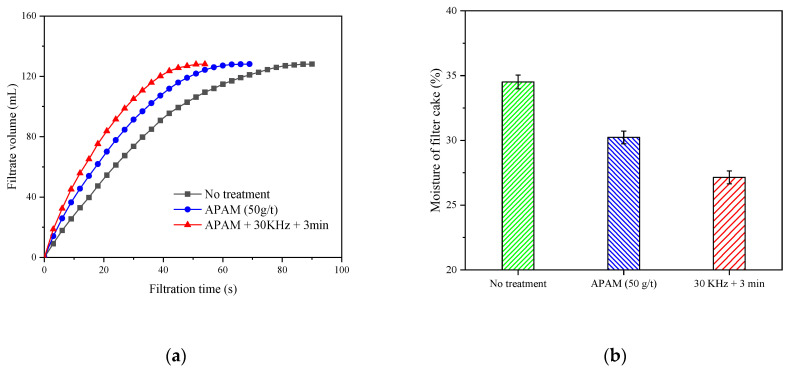
The filtration speed curve (**a**) and the filter cake moisture content (**b**) of different conditions.

The trend of filtrate volume change with increasing filtration time is shown in Figure 8a. The variation of the slurry filtration rate could be reflected by the slope of the curve, and the larger the slope, it indicated that the filtration rate of the slurry was faster. Clearly, the filtration rate of the slurry was significantly increased after the ultrasonic pretreatment. Figure 8b shows the moisture content of the filter cake under different conditions. The APAM led to a filter cake moisture content of 30.2%, whereas the moisture content of the filter cake decreased to 27.1% after ultrasonic pretreatment. This indicates that ultrasonic pretreatment could reduce the filter cake moisture content.

### 3.2. The LRC Slurry Hydrophobicity Analysis

#### 3.2.1. The Contact Angle Measurement Results

**Figure 9:**
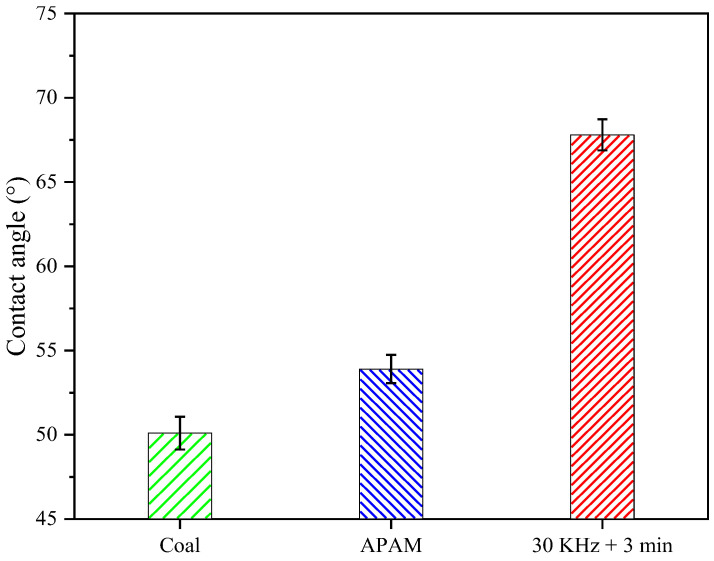
Slurry contact angle under different conditions.

The hydrophilic groups on the surface of LRC was an important factor affecting the contact angle and hydrophobicity of the particles [28,29]. Figure 9 shows the LRC slurry contact angle under different conditions. The coal contact angle was 50.1°, which was increased to 53.9° after the addition of APAM. However, after ultrasonic pretreatment, the contact angle of LRC increased significantly to 67.8°. Since the surface of LRC is abundant in oxygen-containing functional groups—thus, its surface is more hydrophilic—and it is not conducive to the reduction of filter cake moisture [5]. Meanwhile, ultrasonic pretreatment could enhance the dispersion of APAM in the solution environment [30]. This presumed that the ultrasonic pretreatment enhances the interaction between reagents and particles to increase the contact angle and hydrophobicity of the coal surface, thus improving water mobility and promoting filtration.

#### 3.2.2. Viscosity Analysis

**Figure 10:**
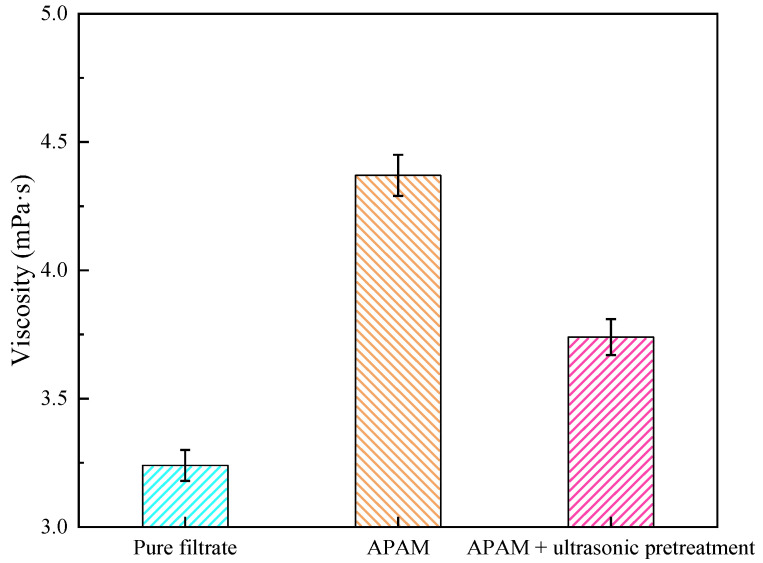
Viscosity change of filtrate before and after ultrasonic pretreatment.

Figure 10 shows the changes of filtrate viscosity before and after ultrasonic pretreatment. The results show that the filtrate viscosity decreased from 4.37 to 3.74 mPa·s after ultrasonic pretreatment. It is speculated that the reason for the above phenomenon is that the ultrasonic pretreatment affected the displacement of APAM molecules and the adsorption of APAM molecules on the particle surface in the coal sludge water system [31]. The decrease in filtrate viscosity leads to an increase in its local Reynolds number, which results in a decrease in drag coefficient and resistance. Therefore, ultrasonic pretreatment helps to reduce the filtrate viscosity and thus improves the coal slurry filtration performance. In addition, it is noteworthy that the viscosity with APAM addition is higher than the filtrate viscosity without APAM addition. This is mainly because APAM is a high-viscosity polymeric organic compound, and its addition increases the viscosity of the filtrate.

#### 3.2.3. XPS Analysis

**Figure 11:**
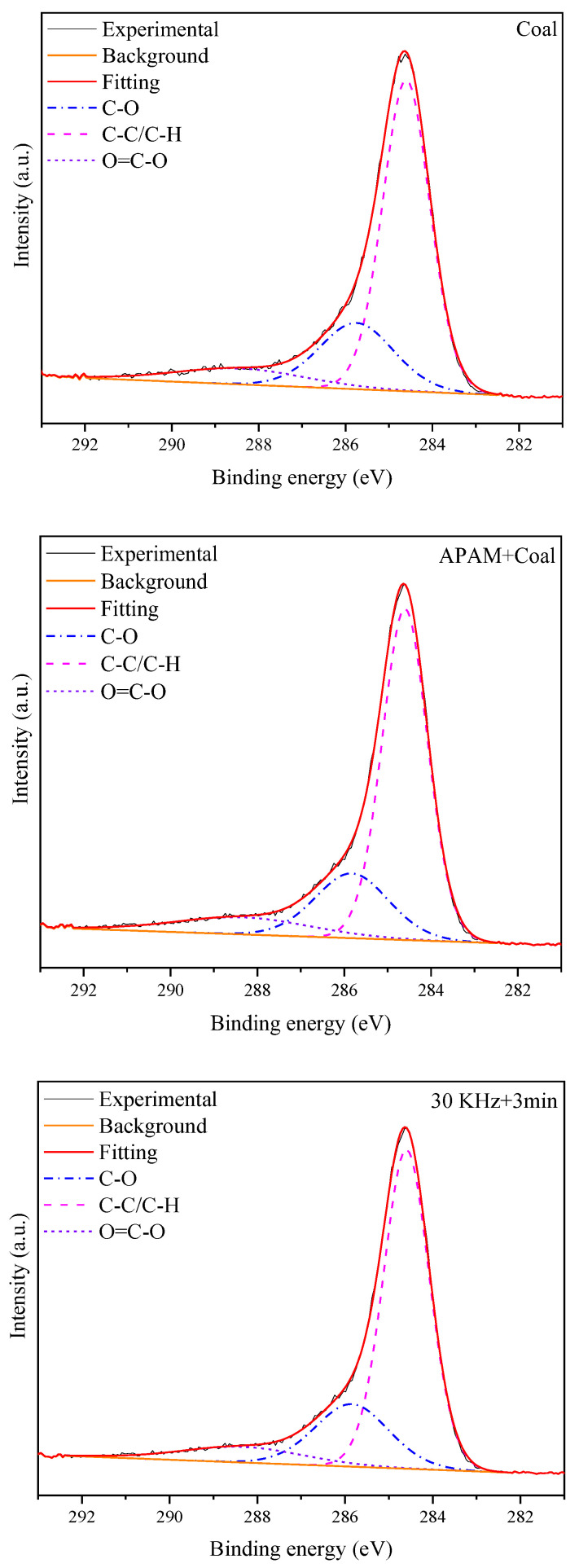
C 1s XPS spectra of LRC slurry under different conditions.

It was shown that the surface hydrophobicity of coal depends mainly on the relative content of hydrophilic and hydrophobic groups on its surface [32,33]. Figure 11 shows the C ls peaks of LRC slurry under different conditions. The groups C-C/C-H, C-O, and O=C-O correspond to peaks at 284.6, 285.6, and 289.1 eV, respectively [34]. Table 3 shows the contents of these three functional groups. The C-C/C-H content of the slurry surface was 68.02% after APAM addition, while the ultrasonic pretreatment increased the C-C/C-H content to 69.22%. Meanwhile, ultrasonic pretreatment reduced the C-O content on the surface of LRC slurry to 21.79% and the O=C-O content to 8.99%. It is assumed that this could be due to the ultrasonic pretreatment enhancing the adsorption capacity of APAM, which resulted in an increase in the hydrophobic strength of the coal surface. This is similar to the conclusion reached by A. Ozkan et al. [35], in which the ultrasonic treatment improved the adsorption of reagents on the colemanite surface and improved the filtration performance. Meanwhile, it is consistent with the contact angle test results.

#### 3.2.4. Surface Tension Analysis

**Figure 12:**
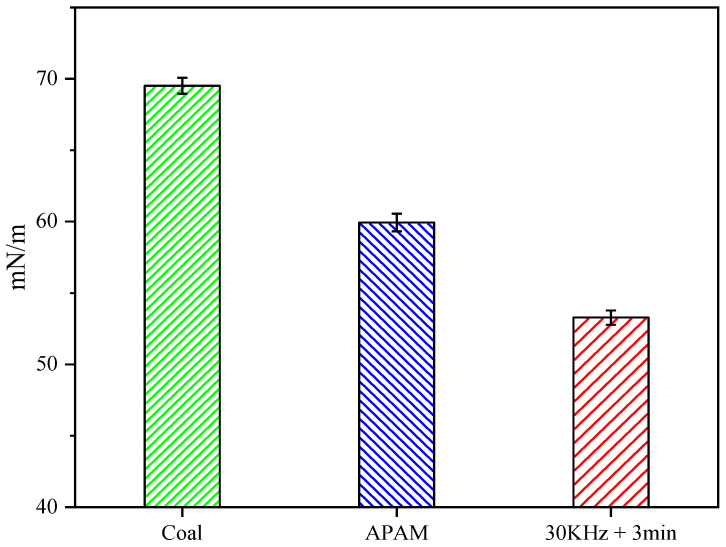
Surface tension of the filtrate under different conditions.

The surface tension of the filtrate affects the rheology and filtration resistance of the filtrate, thus affecting the coal slurry filtration [27,36]. Figure 12 illustrates the changes of the filtrate surface tension under different conditions. The results showed that ultrasonic pretreatment reduced the filtrate surface tension. The filtrate of pure LRC slurry showed a surface tension of 69.5 mN/m. The surface tension of the filtrate was reduced by APAM to 59.9 mN/m. Meanwhile, the surface tension of the filtrate decreased to 53.31 mN/m after ultrasonic pretreatment. The filter cake pore structure could be seen as many aggregations of capillaries with different pore sizes [37]. Meanwhile, the decrease in surface tension of the filtrate can improve the rheology of the filtrate and reduce the filtration resistance of coal slurry, which facilitates the removal of water from the filter cake [5].

### 3.3. The Analysis of Electrostatic Repulsion between LRC Slurry Particles

#### 3.3.1. The Zeta Potential Test Analysis

**Figure 13:**
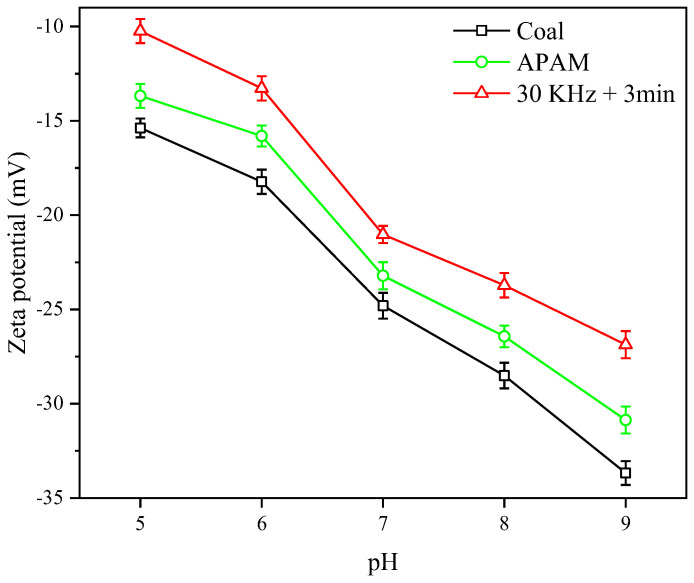
Changes of the slurry zeta potential at different pH’s.

In slurry flocculation filtration, electrostatic repulsion between coal particles was the major force that inhibited particle flocculation [38]. The variation of the zeta potential was analyzed in Figure 13 under the different pH values. The results showed that ultrasonic pretreatment significantly reduced repulsive force between particles. When the filtrate pH = 7, the zeta potential of pure LRC slurry was −24.8 mV. APAM decreased the zeta potential of the slurry to −23.2 mV. After ultrasonic pretreatment, the zeta potential of the slurry was reduced to −21.0 mV. Generally, the farther the zeta potential value was from 0, the more stable was the suspension system. Meanwhile, the closer the zeta potential value was to 0, the weaker was the electrostatic repulsion between the particles, indicating that the suspension was more prone to flocculation [39,40]. This indicated that the ultrasonic pretreatment enhanced the interaction between APAM and particles, while reducing the particle surface potential and the repulsion between particles, thus contributing to the slurry flocculation.

#### 3.3.2. The Change of Floc Structure Properties

**Figure 14:**
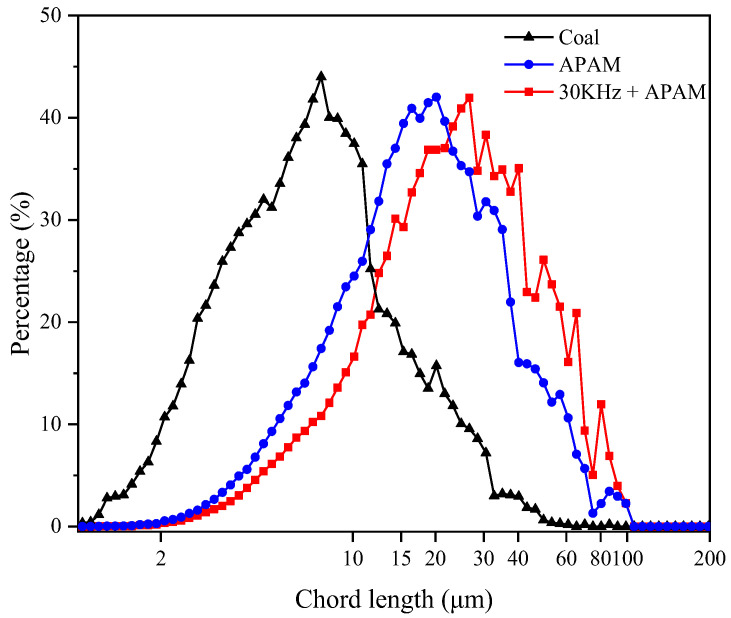
Results of the FBRM test on the chord length distribution of flocs under different conditions.

Flocculation characteristics, such as floc structure strength and floc size, has a significant impact on the cake structure and affects coal slurry filtration [25]. Generally, when the chord length value increases, it indicates an increase in flocculation occurrence and floc size. Conversely, when the chord length decreases, it indicates that the particles in the suspension are uniformly dispersed and no flocculation is occurring [26]. The chord length distribution of the slurry under different pretreatment conditions is shown in Figure 14. Both APAM and ultrasonic pretreatment increase the floc size. The chord length of pure LRC flocs was about 5–10 μm, and APAM increased the chord length to about 15–20 μm. However, the chord length of ultrasonic pretreatment flocs increased to about 25–30 μm. Since APAM is a polymer, it has more branched structures. When it is added to LRC slurry, under the bridging flocculation effect, the particles aggregate with each other to form larger flocs [25]. Meanwhile, the ultrasonic treatment can enable APAM to be uniformly dispersed in LRC slurry. This enables APAM to better agglomerate slurry particles. In addition, zeta potential test results show that the electrostatic repulsive force between slurry particles is reduced, which further promotes the formation of flocs, resulting in the increase in filter cake porosity.

### 3.4. The Filter Cake Properties Analysis

#### 3.4.1. The L-F NMR Test Analysis

**Figure 15:**
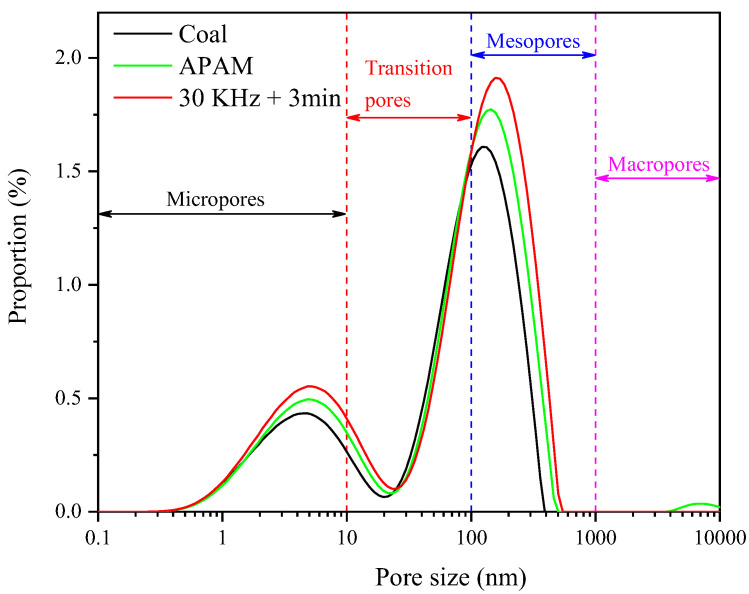
Distribution of the pore size of the filter cake under different conditions.

**Figure 16:**
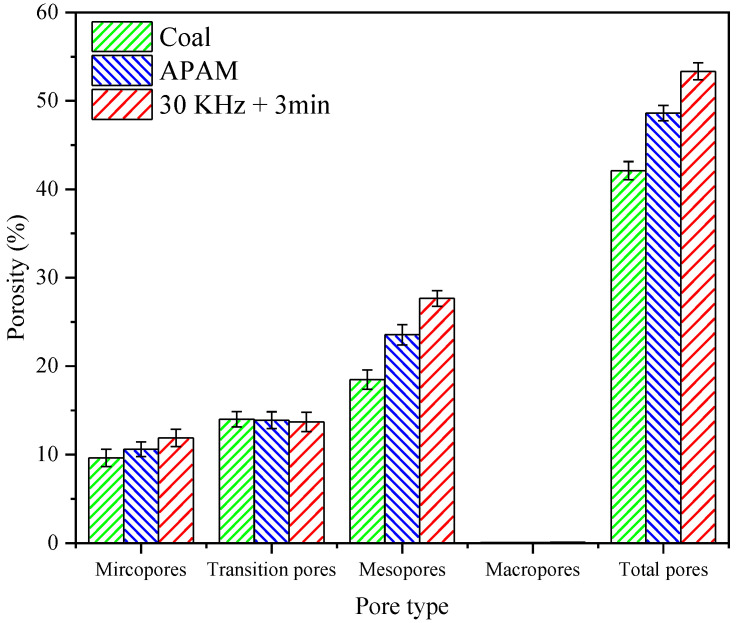
Content of porosity of different types of pores in slurry.

The filter cake porosity is one of the most important factors affecting the filtration rate of coal slurry [24]. The pores of the filter cake, according to the pore size, could be classified as micropores (0–10 nm), transition pores (10–100 nm), mesopores (100–1000 nm), and macropores (>1000 nm) [41]. Figure 15 and Figure 16 show the variation of pore distribution and porosity for different types of pores under different conditions, respectively. The results showed that micropores, transitional pores, and mesopores were the main types of pores contained in the filter cake, while no macropores were found in the cake. The slurry without reagent treatment had a cake porosity of 42.11%, while the addition of APAM increased the cake porosity to 48.61%. However, the filter cake porosity after ultrasonic pretreatment increased significantly to 53.34%, and this increased the filter cake porosity by 11.23%. From Figure 16, it could be seen that the ultrasonic pretreatment has little effect on the micropores and transition pores, but has a great effect on the porosity of the mesopores, which increased by 9.18%. According to the particle size test of the sample, the d_90_ value of the coal sample was 18.975 μm, which indicated that the sample size was fine; thus, the pore types of the filter cake included micropores, transitional pores, and mesopores. APAM promoted the increase in floc size by reducing the repulsion between coals and by bridging flocculation [25,42]. Meanwhile, ultrasonic pretreatment greatly reduced the surface potential of the particles and the electrostatic repulsion between the particles, which increased the floc size and promoted the increase in the filter cake porosity.

#### 3.4.2. Analysis of Filter Cake Characteristics

**Figure 17:**
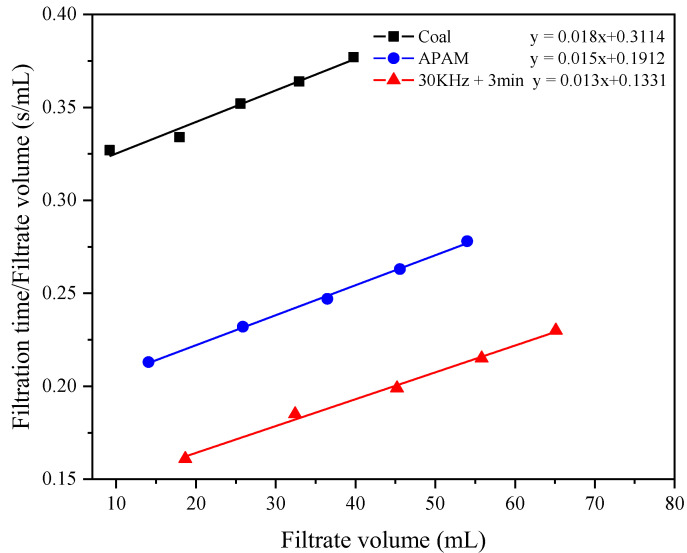
The fitting curve of Equation (2).

Figure 17 illustrates the curve fitted according to Equation (2). To calculate the media resistance (*R_m_*) and filter cake resistance (*α*), the slope and intercept of the curves were used, respectively. Table 4 indicates the filter cake permeability (*K*) calculated by Equation (3). The results showed that ultrasonic pretreatment significantly reduced *R_m_* and *α*. The values of *R_m_* and *α* for pure LRC slurry were 5.621 × 10^−4^ m^−1^ and 3.249 × 10^−12^ m/kg, respectively. However, after ultrasonic pretreatment, the values of *R_m_* and *α* decreased to 3.451 × 10^−4^ m^−1^ and 2.708 × 10^−12^ m/kg, respectively. Meanwhile, the ultrasonic pretreatment contributed to the increase in *K* value. The *K* value was 4.090 × 10^8^ m^2^ without ultrasonic pretreatment, while the *K* value increased to 7.027 × 10^8^ m^2^ after ultrasonic pretreatment. This demonstrated that ultrasonic pretreatment can facilitate the filtration of LRC slurry by decreasing the resistance that the slurry experiences during filtration and increasing the cake permeability.

According to previous tests, ultrasonic pretreatment increased the surface hydrophobicity of the particles, such as increasing the contact angle of coal and decreasing the surface tension. At the same time, ultrasonic pretreatment increased the repulsion between the coals and promoted the flocculation of the particles, thus reducing the electrostatic repulsion between the particles and promoting flocculation, which increased the cake porosity to 53.34%. Among them, ultrasonic pretreatment had the greatest effect on the mesopore porosity, which increased the mesopore porosity by 9.18%. This not only improved the permeability of the cake but also reduced the resistance of the cake. In addition, because the sample contained many fine particles, these particles blocked the probability of the filter media’s pore channel, thus increasing the media resistance. However, ultrasonic pretreatment increased the floc size, which was larger than the pores of the filter media, and the probability of blocking pores was smaller, thus contributing to the reduction of media resistance.

## 4. Conclusions

We investigated the effect of ultrasonic pretreatment on LRC slurry flocculation and filtration. The enhancement of LRC slurry flocculation and filtration performance by ultrasonic pretreatment was investigated from the variations of hydrophobicity, filtrate characteristics, pore structure of the filter cake, and filtration resistance. The following main conclusions were reached.

(1) The ultrasonic pretreatment significantly improved the filtration performance of the LRC slurry; for example, it increased the filtration rate of the slurry and reduced the moisture content of the filter cake. Meanwhile, the ultrasonic pretreatment improved the hydrophobicity of the LRC surface, and it increased the contact angle of the LRC to 67.8° and reduced the filtrate tension to 53.3 mN/m.

(2) Under ultrasonic pretreatment, the zeta potential value of LRC slurry changed from −24.8 to −21.0 mV. In addition, the floc size increased to 25–30 μm. This indicated that ultrasonic pretreatment weakened the electrostatic repulsion between coal particles, thus enhancing the flocculation between particles to form larger size flocs and enhancing slurry filtration performance. 

(3) The porosity of the filter cake increased significantly to 53.34% after the ultrasonic pretreatment. The ultrasonic treatment minimally affected the micropores, transition pores, and macropores. However, the effect on the porosity of mesopores significantly increased the mesopore porosity by 9.18%.

(4) The calculations from Darcy’s filtration theory concluded that ultrasonic pretreatment could significantly reduce the slurry flocculation filtration resistance and improve the permeability. This was mainly because the ultrasonic pretreatment reduced the particle surface potential, thus reducing the repulsive force between the particles and promoting particle flocculation. The increase in coal slurry floc size promotes the formation of a filter cake porous structure and increased porosity, thus decreasing the cake resistance. In addition, the larger floc size was found to have lower probability of blocking the pores of the filter media, thus contributing to the reduction of media resistance.

## Figures and Tables

**Table 1:** The industrial analysis of the LRC sample.

Item	Mad (%)	Aad (%)	Vdaf (%)	FCdaf (%)
Value	7.35	16.74	38.34	54.67

**Table 2:** The parameters used in Equation (2).

*c* (kg/m^3^)	*μ* (Ns/m^2^)	*A* (m^2^)	Δ*P* × 10^6^ (N/m^2^)
200	0.001	0.0095	0.2

**Table 3:** Content of groups on the slurry surface under different conditions.

Conditions	The Content of Different Types of Groups (%)		
	C-C/C-H	C-O	O=C-O
Coal	66.73	22.68	10.59
APAM + Coal	68.02	21.49	10.49
30 KHz + 3 min	69.22	21.79	8.99

**Table 4:** Results of Darcy’s theorem calculations under different conditions.

Project	α × 10^−12^ (m/kg)	R_m_ × 10^−4^ (m^−1^)	K × 10^8^ (m^2^)
Coal	3.249	5.621	4.090
APAM	2.708	3.451	5.528
30 KHz + 3 min	2.347	2.402	7.027

## Data Availability

Not applicable.

## Sample Availability

Samples are available from the authors.
